# A database for oncological research and quality assurance: implementation and first experiences with the University Clinical Cancer Registry Regensburg

**DOI:** 10.1186/s12874-024-02205-6

**Published:** 2024-03-27

**Authors:** Anna Saibold, Michael Koller, Karolina Mueller, Oliver Koelbl, Veronika Vielsmeier, Tobias Pukrop, Oliver Spies, Vivian Eilers, Cathleen Brese, Denise Amann, Julia Maurer

**Affiliations:** 1 Department of Information Technology, University Hospital Regensburg, Regensburg, Germany; 2 Bavarian Cancer Research Center (BZKF), Regensburg, Germany; 3 Center for Clinical Studies, University Hospital Regensburg, Regensburg, Germany; 4 Department of Radiation Oncology, University Hospital Regensburg, Regensburg, Germany; 5 Department of Otorhinolaryngology, Head and Neck Surgery, University Hospital Regensburg, Regensburg, Germany; 6 Department of Internal Medicine 3, University Hospital Regensburg, Regensburg, Germany; 7 University Cancer Center Regensburg, University Hospital Regensburg, Franz-Josef-Strauss Allee 11, 93053 Regensburg, Germany

**Keywords:** Clinical cancer registry, Database software selection, Data migration, Data analysis concept

## Abstract

Legal requirements, certification specifications, as well as the demand for real world data on cancer research and treatment led to the decision to establish the University Clinical Cancer Registry Regensburg. The first organizational step in the implementation process of this oncological data registry was the evaluation and acquisition of suitable tumor documentation and database software. For this purpose, an evaluation matrix comprising required database software criteria was designed and consented by a multidisciplinary group of experts. Next, a yearly report of the Institute for Cancer Center Certification (OnkoZert 2019) was considered to identify database software already in use. The identified systems were rated according to the established criteria matrix and other relevant aspects. Onkostar was the system considered most suited for building up an oncological data repository. In the second step, the central IT department implemented Onkostar on-premise and migrated digitally available data after an adaptation and verification process. In parallel, a uniformed process for handling emerging oncological research questions was established. For research requirements, a data analysis concept was established comprising a proposal for data extraction, procedural instructions, and statistical training materials. In the final step, the implemented software and the process for handling research requirements in practice were evaluated by using two exemplary use cases with the focus on clinic-wide analyses and currently relevant scientific topics. A 2-month test phase conducted by various user groups showed a preference for Onkostar tumor documentation software from IT-Choice, mainly because of its adjustability to support research and treatment. Newly added and migrated data can be used for certification and research purposes. This software also provides support in current tumor documentation by displaying the course of cancer disease for individual patients over time. Such oncological data registries can be a powerful tool for legally required cancer registration, the certification of medical centers, as well as for additional oncological research. Tumor databases can be helpful in projects on cancer treatment and scientific aims. The experiences made at the University Hospital Regensburg may be used as a guidance for implementing clinical databases in similar settings with interdisciplinary responsibilities.

## Background

While the number of annual primary cancer diagnoses has been constantly rising in past decades, survival rates have been drastically improved through new methods of early detection, diagnostics, and therapy [1]. The German National Cancer Plan (Nationaler Krebsplan) [2] has identified four fields for further improving the situation of patients with cancer that include early detection programs, further development of oncological structure supply, quality management, and assurance of efficient oncological treatment with a special focus on patient-centric approaches [1, 3]. In each of these fields, the structured digitalization of oncological data is crucial. Therefore, the newly founded University Clinical Cancer Registry (Universitaeres Klinisches Krebsregister Regensburg, UKKR) set up a database to document all oncological patients treated at University Cancer Center (UCC-R) at the University Hospital Regensburg (UKR). The UCC-R forms the clinical arm of the Comprehensive Cancer Center Ostbayern (CCCO), which as a scientific and clinical facility is the central coordinating institution for interdisciplinary oncological patient care, research, education and training in Eastern Bavaria. As an oncological center of excellence according to the German Cancer Aid Society (DKH) and a maximum care hospital (> 800 beds, 31 departments and institutions), the UCC-R treats all cancer diagnoses (approx. 22,000 patients per year). The CCCO, as a partner in the networks CCCWERA (Comprehensive Cancer Center Allianz Würzburg, Erlangen, Regensburg, Augsburg) and BZKF (Bayerisches Zentrum für Krebsforschung) with its partner sites, is responsible for disseminating its expertise throughout the entire network and thus to peripheral hospitals and oncologists in private practice.

An oncological database has to adhere to pertinent legal requirements. The Bavarian Cancer Registry Law (BayKRegG) [4] requires clinical cancer sites to transmit the pre-defined oncological basis dataset (oBDS) [5] to the Bavarian State Office for Health and Food Safety (LGL) within 2 months after the diagnosis of a cancer disease. The nationwide implementation of clinical cancer registries is aimed at improving cancer treatment. Additionally, the Bavarian Cancer Registry Regulation (BayKRegV) [6] further specifies obligatory transmitted data as oncological basis data, discharge information, and organ-specific data. This regulation also states that the transferal needs to be done electronically or at least in digital format. On top, Book V of the Social Insurance Code (SGB V) § 65c [7] article one indicates that the mandatory registration of cancer disease and its modules has to be done in form of the oncological basis dataset [8, 9].

For the purpose of certification, the oncological basis dataset can be expanded with additional information that is required in the annual certification process. Such required information may be newly diagnosed tumor disease, cancer recurrence, and metastasis. The Bavarian Hospital Law [10] regulates the local collection and analysis of the data of patients treated at a hospital. All additional parameters are transferred in anonymized form and aggregated at the certification institution OnkoZert.

Further data need to be stored for certain research purposes [9]. Any value obtained during cancer treatment may be correlated to treatment outcome in some way. Therefore, it is important to store all additional values together with the minimum dataset in the same database, although these additional values will not be transferred to any national institution. Such storage allows proper data extractions and analyzes to answer research questions and to potentially improve cancer treatment. Research is also legally covered by the Bavarian Hospital Law. The article 27(4) regulates the opportunity for employed practitioners in Bavarian hospitals to use the data available in local hospital databases for intern research projects. The researchers are responsible for the ethically correct and data-protection compliant handling of the data [10]. The necessity of additional data for research and treatment as well as for changing legal requirements led to the decision to establish a registry in Regensburg. The main objectives were (1) fulfilling the legal requirements of cancer registration and certification, (2) complying with the data protection regulation, (3) optimizing data quality through standardization, (4) accelerating documentation, (5) supporting analysis processes, and (6) improving oncological research in particular by improving data interoperability with other subsystems [3, 8, 11].

The aim of this paper is to describe the process of specifying criteria for an oncological database platform, the selection and implementation process, as well as the design of using cases together with a statistical analysis concept. This paper also includes a few recommendations for institutions interested in engaging in a similar process that are based on the made experiences.

## Material and methods

### Selection of appropriate database software

**Fig. 1:**
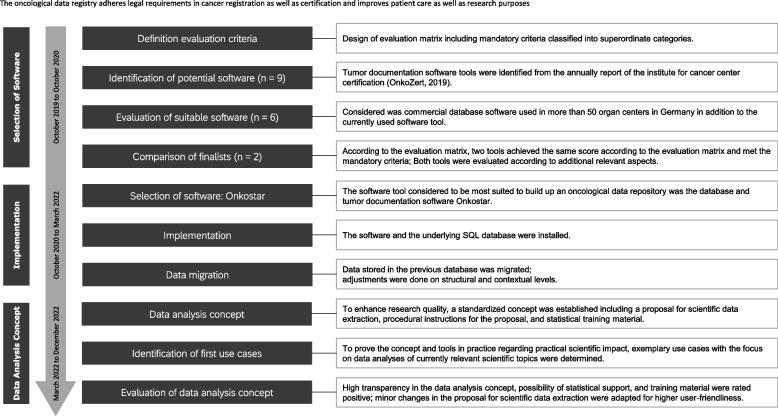
Flow chart—implementation and first experiences with the University Clinical Cancer Registry Regensburg

First of all, a working group was established with selected persons from the fields of clinical cancer registration and information technology to lead the project. The project plan included the steps (1) selection of software, (2) implementation and data migration, and (3) development of a data analysis concept (Fig. 1).

The first organizational step in implementing an oncological data registry was the evaluation and acquisition of an appropriate database together with a software program suited to tumor documentation and registration. Many scientific aspects, for instance, standardization, analysis procedures and data quality, as well as technical aspects such as infrastructure compatibility and data security need to be considered from the very beginning [12].

The yearly report of the Institute for cancer center certification (OnkoZert 2019) was used as a source of potential database software for tumor documentation. To find a database and state of the art software with high usability, sustainability, as well as adaptability, establishment criteria were designed and consented to by an interdisciplinary group of experts (oncologists, information technologists, health care research scientists, data protectionists, data analysists, and UKKR staff) within the UKR. The considered software products were evaluated according to the above criteria.

The required criteria were divided into the following superordinate categories:

1. *Content-related requirements* regarding legally required tumor documentation and certification, i.e. interface of forms as well as data analysis and harmonization with cooperation partners,
2. *Technical and functional requirements* including hardware and software components as well as usability and security aspects, i.e. user management/access rights, interoperability, and data structures, and
3. *Supportive and financial requirements* referring to personnel and license, i.e. maintenance, training, and human resources.

**Fig. 2:**
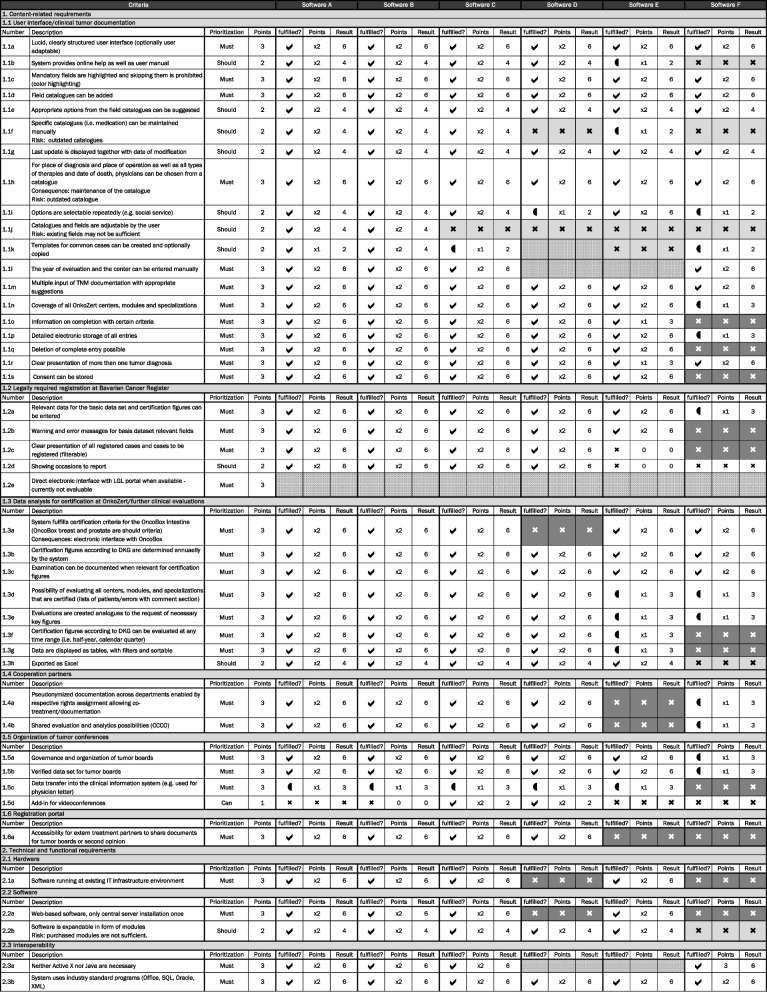

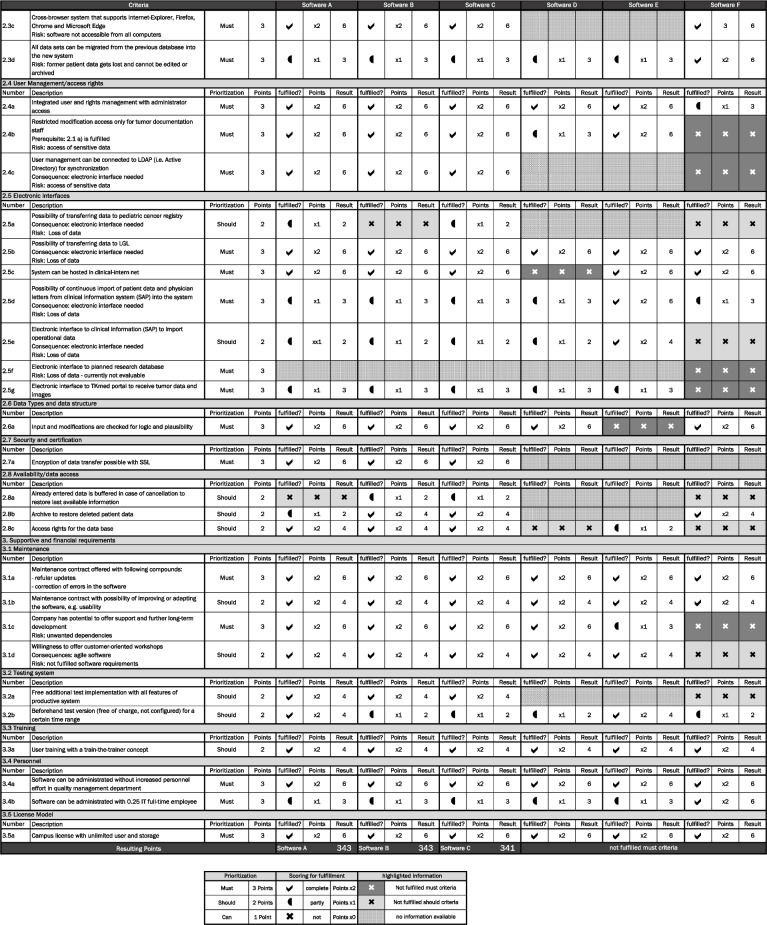
Criteria for evaluating tumor documentation software and evaluation matrix

All required evaluation criteria are described in detail in the evaluation matrix established specific for this project (Fig. 2) and structured into the above-mentioned content-related categories. Each criterion was rated regarding its priority (can = 1; should = 2; must = 3) and degree of fulfillment (not = 0; partly = 1; complete = 2). The product of priority multiplied by fulfilment was calculated for each criterion and a summary score across all criteria was determined. The maximum summary score to be achieved was 382.

### Implementation and data migration

**Fig. 3:**
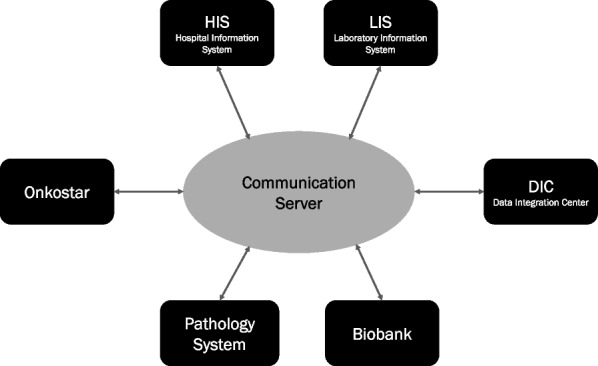
Clinical system environment at UKR

After the most suitable product for building up an oncological database was found, the implementation of hardware and software was started. Virtualized servers were essential for establishing the tumor database and its software into the existing clinical system environment at the UKR (Fig. 3).

The IT department, which is located at the university hospital including specialized representatives for oncological projects, implemented all necessary components, assured the security and provided the application as a web-interface from every internal clinic computer.

For completeness, all digitally available data needed to be migrated into the newly implemented cancer registry database. The data migration process also needed to maintain the correctness of the information. A first set of patient data (approximately n = 60,000) was imported from an existing database that no longer fulfilled the requirements described in the introduction. All oncological data (as required for legal and certification purposes) of patients with a new diagnosis of cancer including therapy, follow-up, and further supplementary data has been documented since January 2021.

### Data analysis concept and first use cases

To get an overview of required or requested projects and necessary adaptations at UKR, demands were informally assessed at all organ-specific cancer centers prior to 2021. Collected data included information about tumor documentation, tumor boards, and other clinic-specific requests, such as molecular pathological documentation beyond the oncological basis dataset.

During the finding period of possible projects, the necessity of a uniformed process for analyzing clinical oncological data of the comprehensive cancer center was recognized. This necessity was indicated on the one side through the rising number of research questions in the field of oncological data and analogously through the rising number of projects and scientific work in this field. Furthermore, the documentation process in general (including accountabilities and responsibilities) and the necessary entity-specific documentation [13] for legally required registration and certification are explicitly regulated by Standard Operating Procedures (SOPs) and are documented in a collaborative knowledge management system that should enhance quality. This system is not part of this paper.

To evaluate the previously elaborated steps and tools in practice, two exemplary use cases with the focus on clinic-wide analyses and currently relevant scientific topics were selected. The evaluation should show the usability of the selected software with its functionalities and adaptations, data migration, and the data extraction process to researching clinicians.

Use case 1 related to the impact of covid-19 pandemic on oncological treatment as part of a retrospective analysis of data of the University Cancer Center Regensburg (UCC-R) in Germany.

Use case 2 dealt with the topic of laryngectomy plus postoperative radio(system)therapy versus primary radio(system)therapy for the treatment of locally advanced laryngeal and hypopharyngeal cancer and presented the results from the University Clinical Cancer Registry Regensburg.

## Results

### Selection of appropriate database software

In March 2020, a set of nine available tumor documentation software systems (n = 9) was extracted from the yearly report (2019) of the Institute for Cancer Center Certification (OnkoZert). Of these nine systems, only commercial database software systems used in more than 50 organ centers in Germany in addition to the currently used software were considered (n = 6).

For the remaining six software systems, termed software A–F in the following text, the evaluation matrix was filled out by a group of representatives with a clinical, technical, or governance background (Fig. 2). Each company of the considered software systems was contacted, and the evaluation matrix was sent prior to the interviews. The interviews were conducted face-to-face in online meetings together with representatives of the software companies and employees specialized on IT applications and system-interfaces. All criteria were discussed one after the other along the evaluation matrix. In parallel, reviews on the different products and databases from other hospitals were obtained.

The software systems E–F did not meet all mandatory criteria and were thus excluded from further consideration. Although software C showed 341 points, it was not considered in the extended evaluation because it lacked the feature of creating own forms; therefore, adjustability was not given. Both, software A and B showed 343 points in total.

In May 2020, a decision was made to further investigate the two software tools A and B by installing test implementations. Additional relevant aspects were formulated.

The most relevant aspects in choosing between these two software systems were information on data input, highlighting of mandatory fields, and plausibility checks. Of similar importance was a clearly structured (especially in case of more than one tumor) and adaptable user interface as well as adaptable selection catalogues (i.e. therapies, physicians, etc.). Additionally the possibility of data input and analysis according to specifications from the DKG (OnkoZert) for all certifiable centers, modules, and specializations (Oncobox, certification figures, etc.) were considerable. A further relevant aspects was the fulfillment of legal requirements regarding the cancer registry law (documentation and registration of the oncological basis data set) including a technical link to the registry portal of LGL and pediatric cancer registry. On top of that, the focus was on the support of scientific analyses in form of structured and clear data extraction (with filters and adjustable to projects) and the possibility of exporting such data and import information such as entries from a previous tumor database. Important aspects were also the organization and accomplishment of tumor boards (together with external cooperation partners) as well as the possibility of archiving and deleting cases and storing consents. The system should also fulfill the technical requirements to be implemented in the existing IT infrastructure (e.g. web-based, industry standards, etc.) and should have user and access management, ideally via LDAP. Further considerations included the potential for on-going development in consultation with the users from UKR. A 2-month test phase by various user groups, mainly tumor documentation staff and IT employees, confirmed the preference for Onkostar tumor documentation software and database from IT-Choice. The main reasons for this preference in addition to the evaluation score were (1) the good user experience with the user interface, (2) the adjustability of own forms, and (3) the recommendations from other university hospitals and cancer registries in Germany.

Onkostar (software tool A) was considered the most suitable software for building up an oncological data repository at the UKR because of its high score in the evaluation matrix and the provision of the above-mentioned additional relevant aspects.

### Implementation and data migration

The first priority was the fulfillment of legal requirements and the registration of relevant data for the required tumor entities by enhancing accurate data input. Data necessary for legal purposes need to be exchanged with superordinate cancer registries in Germany. The structure of the dataset and the interface for exchange of XML files is nationwide standardized for full interoperability. This standardized interface allows direct exchange between different institutions as well as between different specialized software systems and allegorizes interoperability with regard to data protection regulations. These features are the basis for unified oncological documentation and comparable data collection and analysis [14].

Onkostar was implemented by central IT department as virtualized servers on-premise. The implementation included one testing and one productive environment, protected particularly by firewalls. The software is accessible to users as a web-interface from each clinic computer via secure hypertext transfer protocol (https). The underlying database structure is a common SQL database on the central database server.

The software interface allows (1) data input, (2) validation, (3) automation, and (4) adaptation. The graphical interface consists of various forms containing certain fields. The entered data can be automatically validated by a plausibility check or by a final validation of the XML file against the pre-defined schema. Such validation exposes missing mandatory fields, invalid entries, or unusual correlations. Automation in the documentation process can be achieved through digital interfaces with systems from outside; the clinical information system, for example, is directly connected to the oncological data repository via HL7 V2 technology. Basic patient information as well as operational procedures are transmitted and stored automatically. Getting information from a proprietary system imported to the database can be done in a semi-automated manner using the import functionality. If important information, for example for specific research, cannot be stored in the available forms, adaptations can be elaborated. Values and catalogues can be added and clustered in sub-categories, while existing forms can be supplemented with additional features. Completely new forms with individual fields can also be developed.

There are three main user groups: (1) documentation team, (2) selected clinical employees, and (3) administrational staff. In January 2021, the user groups were trained in using the software. Over time, minor modifications in authorizations were adapted. The software is primarily used by the documentation team at UKR for inserting and updating data in the data repository. Selected clinic employees can be authorized to access data records relevant to them, i.e. specific forms or projects, but modification of content is restricted to the first user group. The third group, administrational staff, supports the other user groups. Administrational staff may be employees of the IT department and the quality assurance team, who support with technical, content, and process-related input. Administrational staff have unrestricted access to the documentation system as well as to the database.

After the implementation of the database, the data stored in the database operated at the former Tumor Center Regensburg e.V. were migrated. Because of the similarity of the databases, certain data could be transferred automatically [15]. Reasons for data migration were (1) support of the certification process, (2) support of research requests, and general (3) quality assurance. Data were migrated in several migration rounds with structural and manual adaptions. Preparations for the migration process were the analysis of database structure. After final verification of the correct format, all records were loaded into the system.

Adjustments were done on a structural and a contextual level. Necessary structural adaptations were, for example, mapping medical departments and wards to the current prevalent naming conventions as well as assigning the various tumor boards to the different oncological entities according to the ICD Code. The assignments to the oncological centers were done on the basis of the date of foundation and the ICD code. Another necessary structural adaption was completion of missing patient IDs by a systematic search for the name, surname, and birth date in the clinical information system and partially by manual correction and the aggregation of cases. To achieve the highest possible quality of data, the content of the importable entries was reviewed and adjusted at some points. There were several mappings of items, for example, unifying names of substances, studies, chemotherapy protocols, and the assignment categories (chemotherapy, immunotherapy, targeted therapy, hormonotherapy, and others) of substances. Another example is the mapping of the type of pathological preparation to a new convention. Some data were not imported into the new system, either because of the high effort to map those data or due to the low quality of the existing data. For operations longer than 1 year ago, only relevant information was imported, to keep the database lean. Names of the surgeons as well as referring hospitals and physicians were therefore not imported. Only selected laboratory parameters (i.e. tumor markers) and therapies with connection to oncological treatment were imported. For a few data items, all entries had to be reviewed manually and edited carefully. Special therapies (i.e. RFA or TACE) were assigned to forms, and psycho-oncological and social service organizations needed to be allocated to the primary tumor for patients with recurrent cancer.

Since 1992, all digitally available tumor information has been stored and made accessible in the oncologic data registry. Comprehensive and complete data have become gradually available together with the development of various organ cancer centers over time.

### Data analysis concept and first use cases

A data analysis concept was developed for planning and conducting oncological research within the local and legislative regulations, including professional assistance in the field of data analysis. For that purpose, central documents were established in cooperation with the Center for Clinical Studies (Zentrum für Klinische Studien, ZKS) as guideline and support for clinical researchers.

The first established document is a proposal for scientific data extraction from the UKKR. The template of the proposal starts with general information such as the title, declaration of the responsible person/department, purpose of the proposal (retrospective/prospective), and the type of requested data extraction (aggregated/anonymized/pseudonymized). Followed by a detailed description of the requested data (i.e. selection criteria, data load, aggregation criteria, etc.) together with the description of necessary support for statistical analysis (e.g. estimation of number of cases, introduction into statistic software, specific analyses, and support in publishing). A central component of the proposal is a description of the planned project in form of a study synopsis with the following aspects: the primary aim of the study, secondary aim(s) of the study, the study type/design, the population of interest, inclusion/exclusion criteria, the number of patients, primary and secondary end points, biometry, timeline, planned publication, involved departments, and responsible persons from the clinic. The proposal informs the applicant about other relevant valid documents concerning data protection regulations. These documents need to be acknowledged by signature of the applicant. The last paragraph provides space for remarks and feedback from the (UCC-R and the ZKS towards the applicant. Each person involved as well as their tasks and responsibilities are written down together with the finally extracted dataset and any kind of agreed statistical support.

In parallel, a document with procedural instructions was introduced at UKR to support the completion of the above-mentioned data extraction proposal and to concisely display the entire process. The document implies the objective and purpose, normative references, responsibilities including contact information, and the process from the application to the response with the dataset and optional statistical supervision.

To enhance statistical quality of research, training material for descriptive statistics and ordinary analyses was prepared. If required, clinicians can consult a qualified statistician for statistical support.

**Fig. 4:**
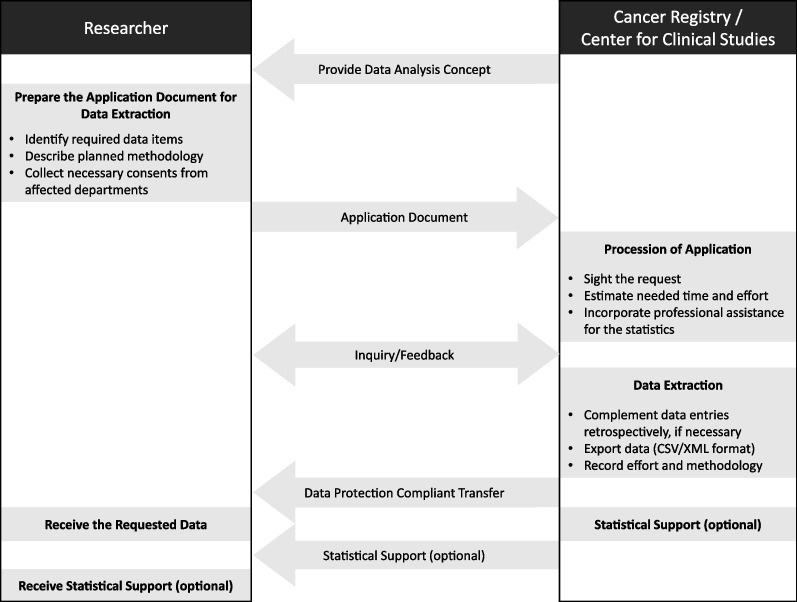
Utilization of the data analysis concept

The concrete steps from the perspective of health care researchers and employees of the cancer Registry and the ZKS are depictured in Fig. 4.

### Use cases

The two use cases differed in data basis and period of time, which shows the broad range of possibilities of the available database and its contents. Tables 1 and 2 summarize the most important characteristics of the use cases and the associations to previously described topics (selection of database, implementation and migration, and data analysis concept).

**Table 1:** Description and evaluation use case 1

Use case	1. Impact of covid-19 pandemic on oncological treatment: retrospective analysis of data of the University Cancer Center Regensburg (UCC-R) in Germany
Description	
Remark/objective	Current topic; across centers; large patient collective; no outcome-analysis
Study design	Only retrospective; no control-group
Data period	Past 3 years (2019–2021); all data since introduction of cancer registry law (2017)
Opportunities	Large number of cases for all relevant centers available in the database Research possibility of the system sufficient to get an overview
Risks/limitations	Several necessary parameters not available in database (e.g. diagnostics); diagnosis often not defined well (distinct point if time, clinical etc.) Fundamentals of documentation not always uniform across centers → Data quality varying throughout centers Oncological basis dataset not sufficient
Evaluation	
Selection of software	No relevant negative aspects Adaptation of covered data items desirable
Database/data migration	No significant impairment through migration due to consideration of only more recent certification-relevant (primary) diagnoses → Already mainly standardized documentation in previous system
Data analysis process	Simple concept, few variables, clear timetable, only retrospective analysis → easy contemplation of proposal document Data extraction easy without connection of forms in the software Statistics (i.e. descriptive, correlations) with training materials practicable

**Table 2:** Description and evaluation use case 2

Use case	2. Laryngectomy plus postoperative radio(system)therapy versus primary radio(system)therapy for the treatment of locally advanced laryngeal and hypopharyngeal cancer—results from the University Clinical Cancer Registry Regensburg
Description	
Remark/objective	Center-specific question, though clinic-wide; deeper entity-related data analysis including outcome-analyses
Study design	Part 1 retrospective Part 2 prospective (based on part 1)
Data period	Since 2010 (before cancer registry law, before foundation of centers)
Opportunities	Large number of cases for the investigated center available in database Research possibility of the system sufficient to get an overview
Risks/limitations	Important parameters not available in database (e.g. details about operations, radio-oncology, comorbidities, toxicity etc.) Diagnosis often not well-defined (distinct point if time, clinical etc.) Inconsistent data quality (dependent on date of foundation of center Insufficient oncological basis dataset
Evaluation	
Selection of software	Possibility of adaptation of forms of particular importance
Database/data migration	No loss of relevant data through migration → additionally needed structured data not available in former database
Data analysis process	Variety of variables necessary → completion of proposal possible in total, simplification of certain aspects in the document necessary Data extraction more complex due to need to connect data over different forms Statistics (i.e. descriptive, correlations) with training materials practicable, multivariate analysis with support from ZKS

First empirical experiences with the data extraction process have shown good comprehensibility of the developed documents without any major obstacles, although shortenings may be possible to avoid redundancies. First extractions revealed incomplete patient data from the years before the implementation of the new oncological cancer registry, which required retrospective manual completion, especially for patients who had not been subject to certification. In order to improve prospective data quality and transparency, the process of documentation is managed in great detail in a collaborative knowledge management system. For certain project-based requirements, forms in Onkostar can be extended in order to be able to record additional items prospectively.

First feedback from scientists using the process rated the high transparency as positive and considered the possibility of statistical support through training materials and contacts in the central study center a benefit.

A summary of the above-mentioned project plan in form of a resulting workflow over time is shown in Fig. 1. This workflow can be used as a reference for project management in comparable implementations in respect of the necessary steps, their timeline, and the relevant specialists.

## Discussion

### Selection of appropriate database software

Starting point of the selection process was a self-created evaluation matrix that proved to be a very practical tool. Primarily, the classification of individual items into the above-mentioned item groups enabled the responsible groups to prepare and work through the individual items in a structured and targeted manner. Furthermore, this classification ensured the inclusion of all essential items in the selection process with high comparability. The matrix also served the software companies as relevant tool, particularly with regard to the preparation for the interviews. Another positive aspect of the matrix was the prioritization of the individual items that enabled compromise solutions for individual items (especially for optional and, in some cases, target criteria).

All those aspects not only allowed fulfilling the content-related requirements but also helped keeping to the scheduled timetable.

When deciding on the most appropriate database software, there was a special focus on the adaptation of the product to meet the needs of the oncologic center of excellence. Primarily, the possibility of adjusting existing selection catalogues and of adding new field catalogues as well as customized forms played an important role in decision-making with regard to future scientific requirements. Particularly one software company showed the potential and willingness to further develop their product in collaboration with users from UKR in a detailed and reliable manner.

### Implementation and data migration

Issues occurring during or after data migration were mainly structural differences between the databases. Therefore, it was not possible to incorporate all data items the same way, which would have led to a loss of data to a certain degree.

In this respect, some item packages needed to be migrated in an entity-specific manner in several migration rounds, hence in a very time-consuming process. Furthermore, such item packages often needed to be worked manually. The reasons for this problem were, for example, unidentifiable patients, difficulties to transform data (i.e. laboratory data), or the active decision to leave data out (e.g. external practitioners due to data protection issues). In summary, the migration process was highly complex and took almost 18 months, much longer than the initially expected 3–6 months.

To enhance data quality in the future, entries were validated manually, for example, samples from all organ centers were taken and monitored. For this reason, the processes for data collection and documentation were adjusted to improve available research data in the long-term. Concrete examples are the above-mentioned use cases, which required the retrospective documentation of missing data. The processes may be adjusted continuously to fulfill the aim of constantly increasing data quality.

The newly added and migrated data can be used for certification and research purposes. The data can also be supportive in current tumor documentation as they display the course of cancer diseases for one single patient over time. Making high quality tumor data available to researchers offers an invaluable benefit to research projects [13].

### Data analysis concept and first use cases

The implemented database as well as the collected and verified data in the cancer registry can be used for data analysis and research requests, for example, to better interpret data and improve clinical performance [16]. To improve oncological research supported by a database and professional assistance in the field of data analysis and statistics, a data analysis concept for planning and conducting oncological research was developed in Regensburg. A uniformed process is needed to ensure a valid data source and therefore valid statistics. This process should increase the analysis of oncological data in the hospital as well as in collaborations, leading to more publications with higher transparency regarding methods, participants, and data sources. Furthermore, a uniform process may promote the publication of study results and improve the transparency of project responsibilities and planned statistics. The quality of clinical epidemiological projects can benefit from statistical consultation.

As mentioned in the results section (Tables 1 and 2), certain data items were identified for both use cases. These data items needed to be emphasized in ordinary prospective tumor documentation, even though they are not part of legal cancer registration or required for certification of centers. For these two projects, it was necessary to retrospectively complement data entries. Data migration did not provide any obstacles in either study and can therefore be assessed positively. The application document for data extraction was usable in both cases, although minor changes were made for more user-friendliness. In respect to the required statistical support, the documents were sufficient because the focus was on descriptive statistics and analysis of correlation. Multivariate analysis was supported by the ZKS.

### Future prospects

Implementation of further projects to enhance the processing and quality of patient data in cancer centers and hospital departments is intended.

As already mentioned, the first step of the process was to determine the requirements of the different departments with regard to improving tumor documentation and the interconnectivity between information systems. Therefore, content-related adaptations (e.g., the addition of specific items) are planned as well as the connection of systems, including structured data import from the clinical information system on the one hand and attachment of the tumor boards on the other hand. Tumor boards face both obligations as well as opportunities, i.e. data organization and documentation on a daily basis but also improved quality in patient care through the complementation of data entries by means of already available information [9, 11, 12].

To further improve the quality of the database, implementation of a module for patient-reported outcome (PRO) is initiated and its expansion arranged. Relevant examples of questionnaires are palliative screening (i.e. IPOS questionnaire) and quality of life based on a standardized set of questions (e.g., EORTC assessment tools). Naturally, synchronized feedback is transferred to the primary clinical information system. The interconnectivity of data together with clinical parameters not only results in tremendous opportunities for the treatment of patients in clinical routine but also for research [9, 11, 12].

High compatibility and interoperability of data can contribute to the success of internal and external research projects. One ongoing local project is the digitalization of genetic and molecular data used in oncology to gain insights into the course of diseases and potential prognoses [9]. There is a necessity for joint projects with interoperable (technically and content based) molecular pathological data items, not only locally but also among oncological centers for example in the scope of the German Network of Personalized Medicine (DNPM) [12, 17].

At the same time, the connection to the newly established data integration center at UKR is in planning. This connection will allow direct exchange with other centers and national initiatives in the trending standard Fast Healthcare Interoperability Resources (FHIR) [18].

The interconnectedness of cancer databases enables the generalizability of research results and the sharing of a voluminous amount of oncological data as well as concepts and standards among different centers. Studies with larger data samples from a larger area will improve regional clinical performance and potentially even healthcare concepts [12, 17]. Harmonization with cooperation partners can be contemplated because sharing the same database structure may lead to benefits when it comes to shared data analysis. In the local network of oncological centers, several institutions are using the same software. The same benefit applies to harmonizing processes and data structures with local partner hospitals. A monthly exchange with partners regarding questions about the actual documentation and quality aspects has been organized. The clinical cancer registry offers enormous opportunities for scientific research, given that harmonization as well as interoperability of clinical studies and screening programs are being continuously improved [9, 16, 19].

### Lessons learned

In general, clinical databases are associated with advantages such as providing a structured format for reviewing patient information, although their development and implementation can be challenging and requires comprehensive considerations [13]. By describing this process exemplary for the UKR, insights may be gained for future implementations of oncological databases in similar settings.

Based on our experiences, it is highly recommendable to start off with a project plan, specify the aims by designing an evaluation matrix, think in terms of statistical analyses plans, and use cases at a very early stage of project development. It also needs to be pointed out that the migration of data from an existing database to a newly established documentation software is a very time-consuming process.

## Conclusion

The software Onkostar was considered the most suitable system in terms of legal requirements as well as certification and research purposes. This highly adaptable and user-friendly tumor database supports research projects as substantiated in the first two use cases. By describing the process of selection and implementation at the University Hospital Regensburg, a guideline has been provided that may be useful for future implementations of oncological databases in similar settings.

## Data Availability

Data sharing is not applicable to this article as no datasets were generated or analyzed during the current study.
